# BLM Sumoylation Is Required for Replication Stability and Normal Fork Velocity During DNA Replication

**DOI:** 10.3389/fmolb.2022.875102

**Published:** 2022-07-01

**Authors:** Christelle de Renty, Kelvin W. Pond, Mary K. Yagle, Nathan A. Ellis

**Affiliations:** ^1^ University of Arizona Cancer Center, University of Arizona, Tucson, AZ, United States; ^2^ Department of Cellular and Molecular Medicine, University of Arizona, Tucson, AZ, United States

**Keywords:** Bloom syndrome, BLM, DNA replication, sumoylation, replication fork stalling

## Abstract

BLM is sumoylated in response to replication stress. We have studied the role of BLM sumoylation in physiologically normal and replication-stressed conditions by expressing in BLM-deficient cells a BLM with SUMO acceptor-site mutations, which we refer to as SUMO-mutant BLM cells. SUMO-mutant BLM cells exhibited multiple defects in both stressed and unstressed DNA replication conditions, including, in hydroxyurea-treated cells, reduced fork restart and increased fork collapse and, in untreated cells, slower fork velocity and increased fork instability as assayed by track-length asymmetry. We further showed by fluorescence recovery after photobleaching that SUMO-mutant BLM protein was less dynamic than normal BLM and comprised a higher immobile fraction at collapsed replication forks. BLM sumoylation has previously been linked to the recruitment of RAD51 to stressed forks in hydroxyurea-treated cells. An important unresolved question is whether the failure to efficiently recruit RAD51 is the explanation for replication stress in untreated SUMO-mutant BLM cells.

## Introduction

DNA replication is a highly coordinated process involving many protein factors. Some factors are stably associated with the replication fork, carrying out functions related to chromosome duplication, whilst other factors associate with the fork when replication is perturbed by DNA damaging agents or inhibitors of polymerases (Dungrawala et al., 2015). Some factors are known to function in both unperturbed and perturbed DNA replication, raising the question whether these factors are responding to damage encountered from physiological levels of DNA lesions generated by normal cellular processes, such as reactive oxygen species, or other sorts of replication limitations that are present in normal, unchallenged cells, such as “difficult-to-replicate DNA,” or perhaps responding to both conditions (Zeman and Cimprich, 2014). The determinants of difficult-to-replicate DNA are not fully understood, but certain types of repeat DNA such as the G-quadruplex-forming DNAs at telomeres provide a potent example. The BLM helicase is one of those factors known to associate with replication forks during unperturbed and perturbed DNA replication (Cunniff et al., 2017), and it represents a tool to better understand the characteristics of difficult-to-replicate DNA.

The BLM gene was identified as mutated in persons affected by the autosomal recessive, clinical entity Bloom syndrome (Ellis et al., 1995). Cells from persons with Bloom syndrome exhibit a striking genomic instability that is characterized by elevated levels of homologous recombination, as evidenced by tenfold higher levels of sister-chromatid exchange and up to fifty times higher levels of quadriradial figures, thought to be the cytogenetic manifestation of mitotic recombination between homologous chromosomes, in appropriately prepared metaphase chromosomes from untreated cells (German et al., 1965; Chaganti et al., 1974). The higher levels of homologous recombination are associated with increased loss of heterozygosity in persons with Bloom syndrome (Groden et al., 1990), which has been associated with increased carcinogenesis, as demonstrated in a hypomorphic *Blm* mouse model (Luo et al., 2000). These correlated factors are likely to be a major contributor to the extraordinary cancer predisposition of persons with Bloom syndrome (German, 1997; Flanagan and Cunniff, 2006).

As an ATP-dependent DNA helicase, BLM prefers substrates resembling replication and recombination intermediates (Bythell-Douglas and Deans, 2020). BLM is complexed with Topoisomerase III alpha, RMI1, and RMI2 (Meetei et al., 2003), and this complex has the unusual capacity to disentangle double Holliday junctions by dissolution (Wu and Hickson, 2003; Raynard et al., 2006; Wu et al., 2006), which led to the interesting proposal that the high sister-chromatid exchange phenotype of Bloom syndrome cells could be accounted for by a default mechanism for double-Holliday-junction resolution that involved breakage and rejoining in the absence of the BLM complex. However, the disentangling activity of the BLM complex can unwind late-replication intermediates (Chan and West, 2018). Moreover, it is also plausible that BLM has a function in synthesis-dependent single strand annealing (Adams et al., 2003), which is the predominant repair pathway for the repair of DNA double strand breaks (DSB) in fruit flies, by directing recombination-associated replication intermediates away from the double Holliday junction pathway.

There is evidence that BLM has a role supporting DNA replication, even though BLM-deficient cells are viable. Indirect immunofluorescence analysis has associated BLM with DNA synthesis in a late-replicating subset of replication forks by co-localization with PCNA and pulse-labelled bromodeoxyuridine (Yankiwski et al., 2000; Zhang et al., 2005). Bloom syndrome cells exhibit defects in Okazaki fragment maturation, as assayed by gel electrophoresis (Lonn et al., 1990), possibly by stimulating flap endonuclease FEN1 activity and prevention of intermediates that stimulate illegitimate homologous recombination (Sharma et al., 2004; Bartos et al., 2006). BLM interacts with the MCM6 subunit of the replicative helicase, and disruption of this interaction leads to supranormal replication fork velocities (Shastri et al., 2021). Replication track analysis of single DNA molecules comparing untreated Bloom syndrome cells to normal cells has shown slower replication fork velocity, increased fork collapse, and increased compensatory dormant origin firing (Davies et al., 2007; Rao et al., 2007). BLM associates with telomere DNA and ribosomal DNA during DNA synthesis, and in BLM-deficient cells, DNA damage accumulates at these genomic locations (Killen et al., 2009; Sfeir et al., 2009; Zimmermann et al., 2014; Drosopoulos et al., 2015), suggesting that BLM has roles at specific, difficult-to-replicate DNA sequences. In *Drosophila melanogaster*, BLM has a critical role in replication of repetitive DNA sequences during the rapid nuclear synthesis phase of the syncytial blastoderm (Ruchert et al., 2022). Finally, analysis of *Xenopus laevis* BLM in the frog oocyte replication system showed that, in the absence of drug-induced replication-associated DNA damage, *X. laevis* BLM associated with replicating chromatin after origin unwinding had occurred, and immuno-depletion of BLM led to the accumulation of DSBs (Li et al., 2004). Accumulation of DSBs in BLM-deficient cells could result from replication defects that lead directly to breakage, or they could be a consequence of under-replication, because BLM has a function in late metaphase disentangling under-replicated segments between sister chromatids (visualized as so-called ultra-fine DNA bridges or UFBs; Chan et al., 2009). Under-replicated segments not resolved by the BLM complex can be resolved instead by breakage and break-induced replication either in metaphase or DSB repair in the next cell cycle in the daughter cell (Minocherhomji et al., 2015; Bhowmick et al., 2016; Spies et al., 2019).

Hydroxyurea (HU) is an inhibitor of ribonucleotide reductase that stalls replication forks by reducing by approximately 50% the levels of dATP and dGTP (Vesela et al., 2017). BLM along with ssDNA binding protein RPA and the recombinase RAD51 are recruited to stalled replication forks immediately after exposure to HU and these proteins accumulate there over time in HU (Dungrawala et al., 2015). The time-dependent accumulation is thought to be due to nascent-strand resection at the fork (Schlacher et al., 2011); however, resection of one-ended DNA breaks could also comprise a part of the total loss of nascent strand DNA. If cells are left in HU for more than 12 h, then stalled forks can collapse, which is defined by replication track analysis as stalled forks that are unable to restart. Accordingly, treatment of cells with HU for 2–5 h or 16 h is an experimental approach to compare responses to replication stress that result in fork stalling or collapse, respectively. It is worthy of note that the propensity of forks to collapse with prolonged HU treatment is species- and cell type-dependent (Hanada et al., 2007; Petermann et al., 2010).

Many proteins associated with the replication fork and active in replication-associated homologous recombination are SUMO substrates (Xiao et al., 2015). We previously found that BLM was sumoylated, predominantly by SUMO2, with preferred SUMO acceptor sites at lysine 317 and lysine 331 (Eladad et al., 2005; Zhu et al., 2008). To address the role of sumoylation in regulation of BLM function, we introduced lysine-to-arginine mutations at amino acids 317 and 331 in a GFP-BLM expression construct and tested for complementation of cellular phenotypes of Bloom syndrome cells. The mutant BLM induced an increase of γ-H2AX foci and diffuse nuclear γ-H2AX in untreated cells, suggesting a mild and chronic defect in DNA replication. Importantly, although spontaneous sister chromatid exchange levels were complemented to normal, RAD51 did not localize with γ-H2AX at sites of replication stress, and we found that RAD51 bound avidly to sumoylated BLM *in vitro*, suggesting sumoylation of BLM is required for efficient recruitment and retention of RAD51 to collapsed replication forks mediated by a SUMO interaction motif (Ouyang et al., 2009; Bergink et al., 2013; Ouyang et al., 2013; Shima et al., 2013). Because RAD51 has a role in maintaining replication fork stability and response to replication damage (Zellweger et al., 2015; Mijic et al., 2017), we hypothesized that replication dynamics, including fork velocity, fork stalling and stability, and fork collapse would be substantially altered in SM-BLM cells. In the present brief report, we examined replication dynamics by replication track analysis and found multiple defects in unperturbed DNA replication and in HU-treated cells that expressed the SUMO-mutant BLM. These results demonstrated that BLM sumoylation is required for normal replication in unperturbed DNA synthesis.

## Material and Methods

### Cell Lines

As described previously (Ouyang et al., 2009), we introduced into the Bloom syndrome cell line GM08505, which is an SV40-transformed fibroblast cell line that has no detectable BLM by Western blot analysis, GFP-BLM expression constructs that encoded either a normal BLM or a BLM with K317R and K331R mutations. We will hereafter refer to cells that expressed the GFP-BLM construct as BLM+ cells and cells expressing the GFP-BLM K317R/K331R construct as SM-BLM cells, for SUMO-mutant BLM-expressing cells. BLM+ cells are fully complemented for all the cellular phenotypes of Bloom syndrome cells that we have scored so far, including the high sister chromatid exchange phenotype. Cells were cultivated as previously described (Eladad et al., 2005).

### Microfluidic-Assisted Replication Track Analysis

Microfluidic-assisted replication track analysis (maRTA) was performed as previously described (Sidorova et al., 2009; Pond et al., 2019). Briefly, DNA was prepared in agarose plugs, DNA stretching was performed on 3-aminopropyltriethoxysilane coated slides (LabScientific) with polydimethylsiloxane (PDMS) capillary microchannel molds, and immunodetection and image acquisition of iododeoxyuridine- (IdU), chlorodeoxyuridine- (CldU), and ssDNA-labeled DNA molecules were performed as previously described (Pond et al., 2019). Only replication signals located on intact DNA fibers were selected for analysis. The maRTA experiments were performed twice on each cell line.

For assessment of fork restart, cells were pulse-labelled with 100 µM IdU for 40 min. Cells were then treated or not with 2 mM HU for 5 h and subsequently released into fresh medium containing 100 μM CldU for 40 min. Signals corresponding to fork restart (dual labelling), fork collapse and termination (IdU label only), and new origin firing (CldU label only) were scored and expressed as a percentage of total signals. For measurement of fork velocity, fork density, and IdU/CldU ratio, cells were sequentially pulse-labelled with 100 μM of IdU and CldU for 40 min.

IdU/CldU ratio were calculated as the ratio of the longest track over the shortest, thus all ratios are ≥ 1 (Techer et al., 2013). Fork density/Mb was calculated by dividing the number of active replication forks by the total length of DNA fibers analyzed, normalized to the fraction of cells in S phase, assessed by flow cytometry (Bialic et al., 2015). Over 300 Mb of total DNA length was measured per condition, and these measures were normalized to percentage of cells in S phase. Signals were measured using NIH Image J software and the data analyzed in GraphPad Prism. Statistical analyses were performed in GraphPad Prism. A Mann-Whitney test was performed to determine statistical significance for fork velocity and IdU/CldU ratio; a Two-Way ANOVA test was performed to test stalled fork restart and collapse, and Ordinary One-Way ANOVA was performed to test variation in fork density.

### Fluorescence Recovery After Photobleaching (FRAP)

BLM+ or SM-BLM cells were treated with 2 mM HU for 24 h and imaged using Leica SP5-II confocal microscope using a 40x/1.25NA PL Apo (oil) lens. Single-foci regions of interest (ROIs) were photobleached using a VIS Ar-488 laser. Recovery was obtained for 1 minute following bleaching at 0.5 s intervals. Time to half of maximum recovery was calculated and represented as T_1/2_. Error bars represent SEM across three independent experiments.

### Western Blot Analysis

Cells were lysed in RIPA buffer supplemented with 5 mM EDTA, 1 mM EGTA, 25 mM sodium fluoride, 1 mM sodium orthovanadate, 1 mM phenylmethane sulfonyl fluoride (PMSF) with 1x EDTA-free protease inhibitor (GoldBio ProBlock Gold; GB-108–2). Protein concentration were measured using Pierce 660 nm Protein Assay. 15 μg of total protein from cell lysates were separated by electrophoresis through Bio-Rad 4%–15% acrylamide mini-PROTEAN TGX gels or Bio-Rad Criterion 10% acrylamide TGX gels, and transferred onto Amersham™ Protran™ 0.45 μm nitrocellulose membranes by semi-dry transfer. To control for protein loading and transfer, prior to blocking, the membranes were stained with Ponceau S (Sigma P7170) for 5–10 min, washed two times with 1% glacial acetic acid in water, and imaged. Membranes were blocked for 1 h in either Tris-buffered saline (TBS) with 5% Bio-Rad Blotting-Grade Blocker, or Prometheus OneBlock Western-CL Blocking Buffer, then incubated with primary antibody in 5% BSA in TBS + 0.1% Tween-20 overnight at 4°C. After washing with TBS-T the membranes were incubated with secondary antibodies (HRP-linked anti-mouse, Cell Signaling Technology 7076 or HRP-linked anti-rabbit, GE Healthcare NA934 V); imaging was done on a Syngene G: Box Chemi gel documentation system. Primary antibodies for immunodetection were obtained as follows: anti-BLM (Beresten et al., 1999), anti-CHK1 (Cell Signaling Technology; 2345), anti-phospho-CHK1 (Ser345) (Cell Signaling Technology; 2348), anti-RPA2 (Abcam; ab2175), anti-phospho-RPA2 (Ser4/8) (Bethyl; A300-245 A), and γ-H2AX (Cell Signaling Technology; 9718).

## Results

### SUMO-Mutant BLM Failed to Restart Some Stalled Replication Forks

**FIGURE 1:**
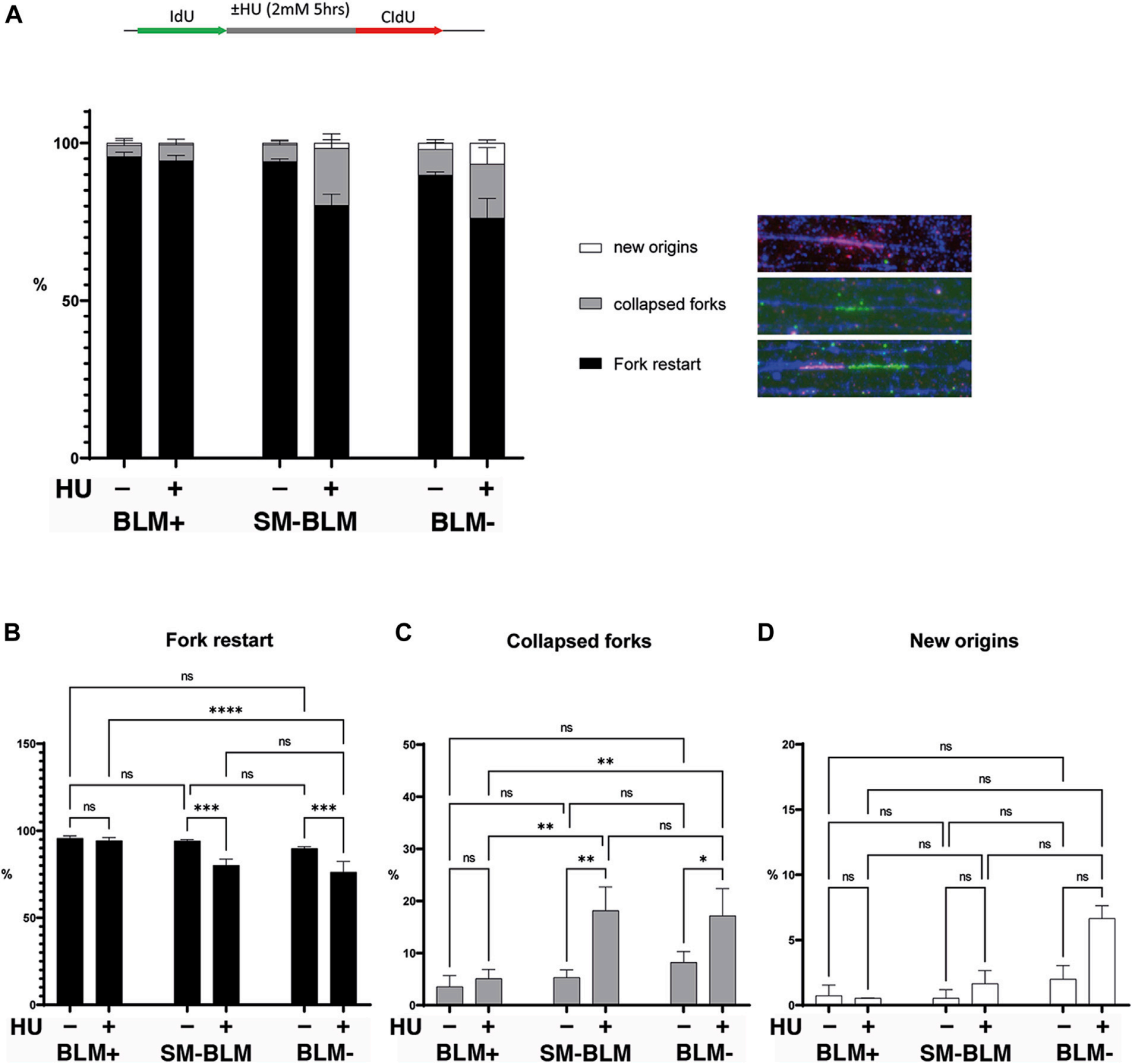
SUMO-mutant BLM failed to restart some stalled forks under replication stress. As shown in the schematic image, cells were pulse-labelled with IdU, followed or not by a 5-h treatment with HU, and finally released into media containing CldU, after which maRTA was performed. **(A)** Signals corresponding to fork restart, collapsed forks, and new origin firing were scored and expressed as a percentage of total signals. Over 600 signals per condition were analyzed. Two independent experiments were performed, and mean and standard deviation are shown. Data were analyzed using ordinary two-way ANOVA followed by Tuckey’s multiple comparison test Corresponding statistical significances are indicated for each group plotted individually: fork restart **(B)**, collapsed forks **(C)**, and new origins **(D)** (ns, not significant; *, *p* < 0.05; **, *p* < 0.01; ***, *p* < 0.001; ****, *p* < 0.0001). Full statistical analysis is provided in Supplementary Table S1 and primary data is provided in Supplementary Data S1.

BLM sumoylation is required for repair of collapsed forks (Ouyang et al., 2009) and for licensing of HR repair factors upon fork stalling (Ouyang et al., 2013), suggesting that BLM sumoylation promotes fork stabilization upon replication stress. Using maRTA, we assessed the ability of stalled forks to restart after replication stress. After 5 h of HU treatment, in BLM+ cells, most of the stalled replication forks were able to restart (Figures 1A,B), as previously described for those conditions (Petermann et al., 2010). Consistent with previous reports (Davies et al., 2007), *BLM*-deficient cells showed a reduced frequency in fork restart, which was associated with increased fork collapse (Figures 1A–C). Similarly, SM-BLM cells showed a defect in fork restart. Increased fork collapse was associated with increased new origin firing; however, this effect was smaller in SM-BLM cells compared to BLM-deficient cells and did not reach statistical significance, most likely due to the low number of events observed (Figures 1A,D). Together, these results confirmed that BLM sumoylation was required for efficient fork restart upon replication stress.

### SUMO-Mutant BLM Showed Impaired Replication Fork Progression

**FIGURE 2:**
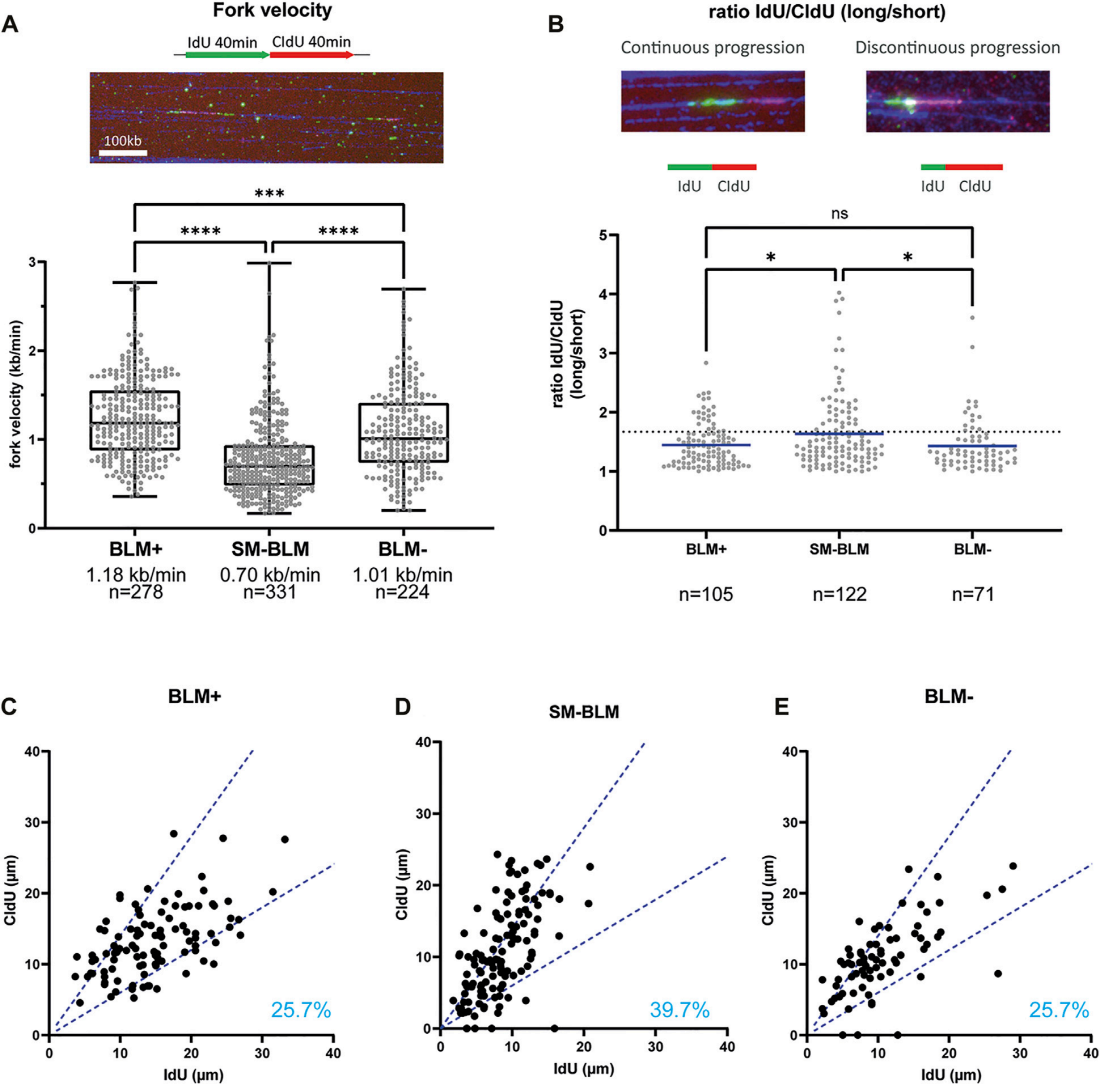
SUMO-mutant BLM showed impaired replication fork progression. **(A)** Cells were labelled by two successive 40 min pulses of IdU and CldU. Intact replication tracks as seen in the picture were measured to calculate fork velocity. The graph shows the distribution of replication fork velocities measured by maRTA from two independent experiments. Box and whisker plots represent 25–75 percentiles and minimum and maximum values, respectively. Median fork velocity and total number of signals is indicated for each cell line. **(B)** Distribution of IdU/CldU ratio of active replication forks from two independent experiments. Ratios are expressed as the ratio of longer track over the shorter track, regardless of halogenated nucleotide for which an excess value was obtained. Mean is indicated. Dotted line represents 40% difference between IdU and CldU length. Data was analyzed using Mann-Whitney non-parametric test (ns, not significant; *, *p* < 0.05; **, *p* < 0.01; ***, *p* < 0.001; ****, *p* < 0.0001). **(C–E)** For each active fork represented in B, length of IdU track was plotted against length of CldU track. Dotted blue lines indicate 40% difference between the two signals. Percentages indicate the percentage of asymmetric labeled track (forks that have more than 40% difference between IdU and CldU track lengths). Primary data are provided in Supplementary Data S1.

BLM plays a role in unperturbed DNA replication and *BLM*-deficient cells exhibit slower fork progression (Rao et al., 2007). In addition, constitutive DNA damage was present in SM-BLM cells, as measured by γ-H2AX foci and RPA foci (Ouyang et al., 2009; Ouyang et al., 2013), which suggested these cells experience chronic perturbations in DNA replication in the absence of exogenously induced DNA damage. Consequently, we measured replication fork progression under unperturbed conditions using maRTA. SM-BLM cells exhibited a median fork velocity of 0.70 kb/min compared to 1.18 kb/min in BLM+ cells (Figure 2A). As previously described, *BLM*-deficient GM08505 cells also exhibited reduction of fork progression, with a median of 1.01 kb/min (Figure 2A). Together, these results indicated that BLM sumoylation was required for normal DNA synthesis in physiologically normal conditions.

To determine if this slower fork progression was associated with increased firing of replication origins, global fork density was analyzed (Table 1). We found that fork density was increased in BLM-deficient GM08505 cells (2.0 forks/Mb), similar to what was found in previous reports (Davies et al., 2007; Rao et al., 2007). Fork density in SM-BLM cells (1.9 fork/Mb) was also higher compared to BLM+ cells (1.2 fork/Mb). The higher fork density found in SM-BLM cells indicated that SM-BLM cells fire more origins of replication than BLM+ cells.

**TABLE 1:** DNA replication fork density (forks/Mb DNA).

		BLM+	SM-BLM	BLM-
Experiment 1	Normalized total DNA (Mb)^a^	126.21	114.26	127.80
	Total forks	162	207	263
	No. forks/Mb	1.3	1.8	2.1
Experiment 2	Normalized total DNA (Mb)^a^	262.29	133.36	156.72
	Total forks	276	271	292
	No. forks/Mb	1.1	2.0	1.9
**AVERAGE**	**No. forks/Mb**	**1.2**	1.9*	2.0*

BLM+, cells that express a normal GFP-BLM protein; SM-BLM, cells that express a SUMO-mutant GFP-BLM protein; BLM-, the parental GM08505 SV40-transformed fibroblast cell line derived from a person with Bloom syndrome. No. fork/Mb is a measure of fork density.

a Over 300 Mb of DNA was measured for each condition and then normalized to the fraction of cells in S phase.

*p < 0.05 by Ordinary One Way ANOVA, comparing SM-BLM or BLM- to BLM+.

Shorter replication tracks could reflect increased fork stalling. To assess fork stalling, we measured IdU/CldU ratio of active replication forks (Figures 2B–E). If replication machinery is continuously progressing, the length of IdU and CldU tracks should be similar (ratio ∼ 1). However, if replication machinery is encountering obstacles during one of the pulses, this ratio will deviate from one. IdU/CldU ratio in SM-BLM cells indicated an increase of fork stalling (Figure 2B). Nearly 40% of forks (39.7%) exhibited an asymmetry between the two tracks (Figure 2D) compared to 25.7% for BLM+ cells (Figure 2C). Interestingly, based on this parameter, BLM-deficient GM08505 cells exhibited similar levels of fork stalling compared to BLM+ cells (Figures 2B,E), which was inconsistent with previous results reporting increased fork stalling in BLM-deficient cells as measured by sister-forks asymmetry (Rao et al., 2007). In summary, the slower velocity of replication forks and the increased IdU/CldU ratio in SM-BLM cells compared to BLM-deficient cells were consistent with a more severe DNA replication defect in SM-BLM cells.

### Slower SUMO-Mutant BLM Turnover at Stalled Replication Forks

**FIGURE 3:**
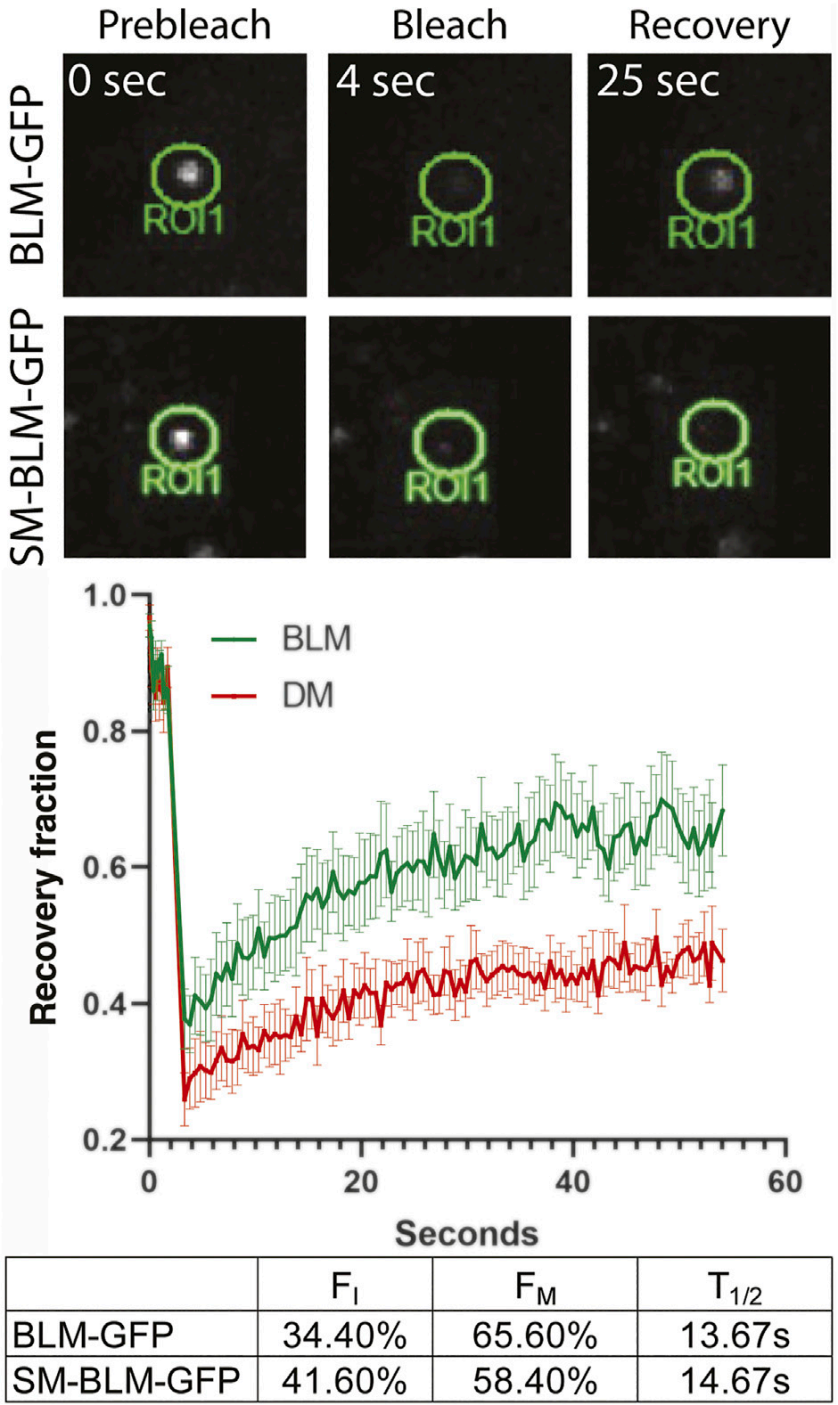
Mobility of SUMO-mutant BLM at stalled forks is reduced. BLM + or SM-BLM cells were treated for 24 h with 2 mM HU. Single-foci regions of interest (ROIs, i.e., focal accumulations of GFP-BLM protein) were photobleached and signal recovery was followed for 1 min after bleaching at 0.5 s intervals. Examples of bleached ROIs are shown (top), and recovery fraction over time was plotted (bottom). Error bars represent SEM from three independent experiments. Time to half of maximum recovery is shown as T_1/2_. F_I_ and F_M_ represent immobile and mobile fractions, respectively.

Sumoylation is a dynamic process, allowing protein-protein interaction, modulating protein function, and promoting protein turn-over (Hay, 2005). Study of the yeast homolog Sgs1, suggested that BLM is most likely recruited to and retained at stalled forks through its SUMO interaction motif by interaction with a sumoylated form of the SMC5/6 complex (Eladad et al., 2005; Bonner et al., 2016). Once recruited to the SMC5/6 complex, BLM is sumoylated by the NSMCE2 SUMO E3 ligase component of that complex (Pond et al., 2019). We have shown previously that SUMO-BLM is a substrate for SUMO-targeted ubiquitin ligase (STUbL) RNF4, suggesting that sumoylation may be required for BLM turnover at stalled and collapsed forks (Ellis et al., 2021). Consequently, we asked whether BLM dynamics at collapsed replication forks were affected by sumoylation, by performing fluorescence recovery after photobleaching (FRAP) at sites of replication collapse in BLM+ and SM-BLM cells generated by treatment with 2 mM HU for 24 h (Figure 3). Following photobleaching of GFP-BLM foci, signal recovery of SM-BLM was significantly slower compared to recovery in BLM+ cells (Figure 3). In addition, the immobile fraction of SUMO-mutant BLM is increased to 41.60%, compared to 34.40% for normal BLM. These results indicated that SM-BLM is less mobile at sites of replication stress in the absence of sumoylation. We concluded that sumoylation facilitates normal BLM turnover at stalled and collapsed replication forks.

### ATR Not Activated by SM-BLM in Unperturbed Cells

**FIGURE 4:**
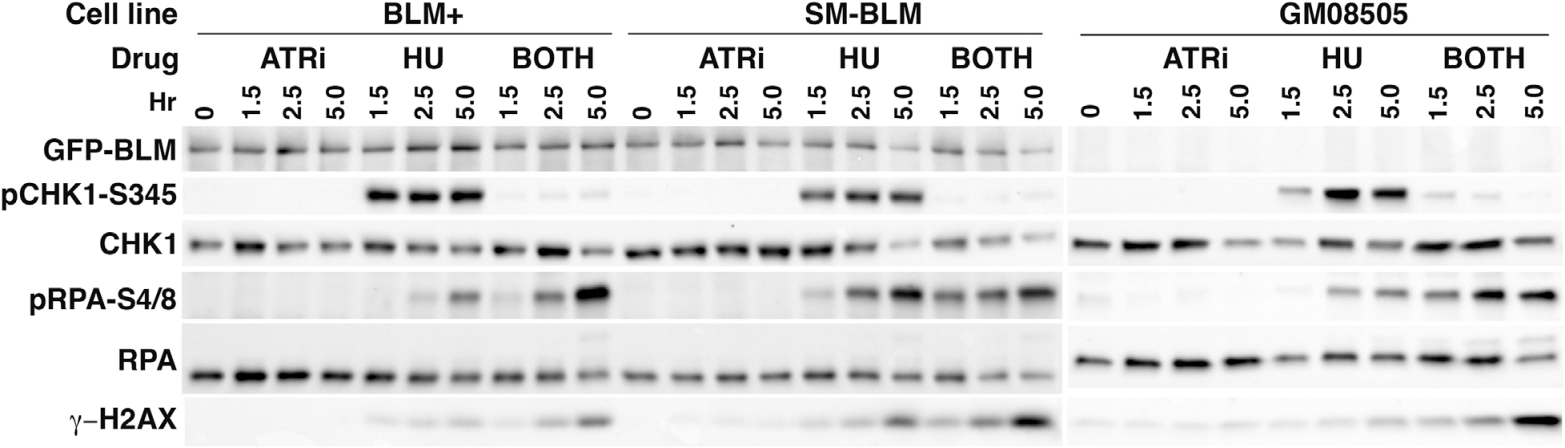
CHK1 phosphorylation is normal in SM-BLM cells. BLM+, SM-BLM, and BLM-deficient GM08505 cells were treated with 2 mM HU for 1.5, 2.5, and 5 h or left untreated. Cell lysates were prepared and analyzed by Western blot with antibodies to the indicated proteins. Proteins in the BLM + and SM-BLM lysates were separated by SDS-PAGE through Bio-Rad Criterion 10% acrylamide gels and proteins in the GM08505 lysates were separated through Bio-Rad 4%–15% acrylamide mini-PROTEAN gels.

ATR is activated during replication stress, and it regulates the firing of dormant origins and the exposure of ssDNA in response to HU treatment. We sought evidence for increased replication stress in unperturbed SM-BLM cells by analysis of CHK1 phosphorylation by Western blot. Concomitantly, we measured the extent of fork damage in HU-treated cells in the presence and absence of ATR inhibitor by measuring RPA hyper-phosphorylation at serine 4 and 8 and γ-H2AX. No increase in CHK1 phosphorylation was evident in SM-BLM cells in the absence of HU treatment, suggesting that the extent or nature of replication stress under physiologic conditions in these cells does not result in ATR activation (Figure 4). In contrast, as we have reported previously (Ouyang et al., 2009; Ouyang et al., 2013), HU induced excess levels of RPA phosphorylation and γ-H2AX in SM-BLM cells compared to BLM+ cells, and inhibition of ATR exacerbated these effects more in SM-BLM cells, suggesting that SUMO-mutant BLM and ATR effectively acted additively at stressed replication forks.

## DISCUSSION

Protein sumoylation is a highly dynamic process in which, under the appropriate conditions, a large fraction of the pool of a protein can be sumoylated, but the steady-state levels of sumoylated protein may exhibit a very limited change. Sumoylated proteins can be difficult to detect because desumoylation activities of SUMO proteases (SENPs) and SUMO-mediated protein degradation through the ubiquitylation activity of RNF4 keep the steady-state levels of sumoylated protein low. Based on Western blot analysis of cell lysates and immunoprecipitated BLM, under uninduced conditions the steady-state levels of sumoylated BLM are less than 2% of total BLM protein (Eladad et al., 2005; Zhu et al., 2008), but with HU treatment they can increase several fold yet still be difficult to detect. Nevertheless, cells that express SM-BLM protein exhibit striking defects in replication fork velocity, IdU/CldU ratios, and the frequencies of HU-induced collapsed forks that are at least as severe or more severe than what is seen in BLM-deficient GM08505 cells. In fact, the unique phenotypic effects associated with expression of a SUMO-mutant form of BLM—including the increased levels of focal and diffuse γ-H2AX, the high levels of RPA foci in untreated and HU-treated cells, and the failure to properly recruit and retain RAD51 to collapsed forks—very likely trace their origin to defects in normal progression of the replication fork. Sumoylation was shown here to be an essential part of BLM’s function at DNA replication forks in both unperturbed and perturbed DNA synthesis. Although sumoylation has been previously implicated in normal DNA replication in studies of induced replication stress, to our knowledge, the present results are the first demonstration of a strong dependence on a specific sumoylation event for unperturbed, physiologically normal DNA synthesis.

Our data suggested fork stalling is more severe in SM-BLM cells compared to BLM-deficient cells based on the γ-H2AX, RPA, and RAD51 phenotypes and based on our measure of fork instability using the IdU/CldU ratio. However, Rao et al. (2007) measured fork instability using fork asymmetry, that is, testing symmetry between two forks emanating from a single origin. We did not see a difference in IdU/CldU ratio in GM08505 cells, but Rao et al. observed sister-fork asymmetry in the same cell line. Perhaps the IdU/CldU ratio test is a less sensitive test for a fork-stalling defect than sister-fork asymmetry. Alternatively, IdU/CldU ratio could be more sensitive to the greater fork velocity difference in SM-BLM cells compared to BLM-deficient cells.

In genetic terms, the SUMO lysine to arginine substitutions of BLM at amino acids 317 and 331 are separation-of-function mutations. Sumoylation appears to strongly influence BLM’s associations with two central factors in homologous recombination by diminishing its interaction with RAD51 and increasing its interaction with RPA, without affecting its DNA helicase activity yet increasing its affinity for ssDNA (Ouyang et al., 2009; Ouyang et al., 2013). BLM is known to interact with DNA2 to catalyze resection of 5′ recessed ends of DSBs (Gravel et al., 2008; Nimonkar et al., 2011; Soniat et al., 2019; Whelan and Rothenberg, 2021), and the increased levels of focal RPA after prolonged HU treatment indicates that SM-BLM promotes resection, that is, it is hypermorphic with respect to the fork resection phenotype. A separation-of-function mutation that interrupts BLM’s interaction with RPA has no effect on DSB end resection or on BLM’s and RPA’s association with UFBs; moreover, it complements the high sister-chromatid exchange phenotype (Shorrocks et al., 2021). However, the RPA interaction-negative BLM is associated with increased replication stress, as evidenced by slower fork velocity and lower IdU/CldU ratio, and it exhibits less fork resection, as evidenced by less RPA accumulation at collapsed forks in HU-treated cells. We suggest that the function of BLM sumoylation is to tip the balance away from fork resection and in favor of a more stable interaction with RAD51, which has the salutary effect of fork stabilization at difficult-to-replicate DNAs. The work of Shorrocks and colleagues further suggests that interaction with RPA is also salutary but not in excess as seen with the SM-BLM mutant.

The most striking of the replication defects in SM-BLM cells related to the reduction in fork velocity, which exhibited a more severe phenotype compared to BLM-deficient cells. The FRAP results showed that SUMO-mutant BLM is less mobile at stalled forks, suggesting that sumoylation regulates protein turnover there and possibly relieves some inhibitory activity of the BLM complex. It may also destabilize interactions with protein factors besides RPA. Recently, it was shown that BLM interacts with MCM6 and disruption of BLM interaction with MCM6 results in increased fork velocity in unperturbed cells (Shastri et al., 2021). These results raise the possibility that BLM sumoylation negatively regulates interaction with MCM6, thereby modulating fork velocity in response to replication stress or at sites of difficult-to-replicate DNA.

With regards to the mechanism by which BLM sumoylation facilitates the function of the replisome in unperturbed, physiological normal DNA synthesis, we suggest the following model. There is evidence in yeast that SMC5/6 complex is a sensor of fork slowing or pausing (Menolfi et al., 2015; Peng et al., 2018). SMC5/6 is recruited to forks that encounter difficult-to-replicate DNA, which is formally defined as regions of DNA in which the replisome slows or pauses. Binding of SMC5/6 to the replisome activates the E3 SUMO ligase of the NSMCE2 component of the SMC5/6 complex. The SMC5/6 complex becomes sumoylated, BLM is recruited to the replisome from the PML nuclear bodies, and NSMCE2 sumoylates BLM. Earlier work showed that RAD51 is not recruited to collapsed replication forks in SM-BLM cells (Ouyang et al., 2009) and RAD51 is required to stabilize stalled forks (Zellweger et al., 2015; Mijic et al., 2017). Thus, replication forks that encounter DNA damage or difficult-to-replicate DNA in which the replisome slows or pauses require BLM sumoylation for recruitment of RAD51 and stabilization of the fork. Consequently, in SM-BLM cells, unperturbed replication forks have a propensity to collapse, which induces ATR-dependent dormant origin firing. In BLM-deficient cells, RAD51 gets recruited to stressed forks, suggesting further that the presence of the SUMO-mutant BLM at stressed forks blocks the recruitment of RAD51, whereas in BLM-deficient cells RAD51 can be recruited directly to stressed forks through RAD51’s sumo-interaction motif binding to sumoylated SMC5/6 complex. Future tests of this model in mammalian cells would include complementation of SM-BLM cell defects by independent recruitment of RAD51 to stressed replication forks, further analysis of the SUMO interaction motif on BLM and RAD51, and analysis of separation of function alleles of NSMCE2.

As noted above, SM-BLM was less mobile at stalled forks, suggesting that one role of sumoylation is to control the turnover of BLM during replication stress. Consequently, we might expect that inhibition of the processing of SUMO-BLM by the STUbL RNF4 by siRNA depletion might produce some similar phenotypes by leaving an excess of immobile, albeit sumoylated, BLM protein at sites of damage. We recently reported that SUMO-BLM is a substrate of RNF4 and RNF4 depletion led to a hyper-accumulation of SUMO-BLM at collapsed forks generated by prolonged HU treatment (Ellis et al., 2021). Consequently, we expected that sumoylated BLM would also be less mobile at collapsed forks. To some surprise, we tested GFP-BLM mobility in HU-treated RNF4-depleted cells and did not see a reduction in its mobility (data not shown). Because this is a negative result, we are careful not to over-interpret it. Our previous analysis of the effects of RNF4 depletion were conducted mainly in U2OS cells, and we do not know how much SUMO-BLM accumulates in the SV40-transformed fibroblast cells used in the present experiments upon depletion of RNF4 by siRNA transfection. In support of this concern, the alterations in replication dynamics, namely, the high levels of new origin firing, that we observed in RNF4-depleted U2OS cells were not observed when replication track analysis was carried out in RNF4-depleted BLM+ cells. Further analysis of SUMO-BLM levels in RNF4-depleted cells needs to be carried out, and, for this analysis to proceed, cells expressing a His-tagged SUMO2 must be generated to facilitate detection of low levels of SUMO-BLM.

In a careful examination of other phenotypes, we noted little phenotypic concordance between SM-BLM cells and RNF4-deficient cells. The differences are enumerated as follows (Ouyang et al., 2009; Ouyang et al., 2013; Ellis et al., 2021): 1) Numbers of BLM foci in untreated SM-BLM cells were 2.5 times higher but these numbers were not increased after treatment with HU, whereas BLM foci in untreated RNF4-deficient cells were no different from untreated control and HU treatment increased the median number by over 20%. 2) γ-H2AX focal numbers were significantly elevated in untreated SM-BLM cells, and they increased excessively after HU treatment, but the numbers of γ-H2AX foci were not different from control in untreated or HU-treated RNF4-deficient cells. 3) Focal concentrations of RPA were excessively elevated in untreated and HU-treated SM-BLM cells, but the numbers of RPA foci in RNF4-deficient cells were the same as in normal cells. But, in RNF4-deficient cells, there was instead an increase in the size of RPA foci. 4) There were nearly half as many RAD51 foci in HU-treated SM-BLM whereas the reduction in RAD51 foci in RNF4-deficient cells was not significant. Thus, the immunofluorescent phenotypes for BLM, RPA, RAD51, and γ-H2AX seen in RNF4-deficient cells were not at all similar to the phenotypes observed in SM-BLM cells. 5) DSBs were elevated in SM-BLM cells and not different from normal in RNF4-deficient cells. Finally, 6) the replication dynamics, including fork velocity, fork restart, and fork collapse were not significantly different from control in RNF4-deficient cells treated with HU for 2 hours; instead, a difference was only seen after prolonged HU treatment, and the most notable finding there was an excess of new origin firing relative to collapsed forks. Altogether, the phenotypes of RNF4-deficient cells are different from those of SM-BLM cells, suggesting that there is a meaningful difference between the molecular pathology of SUMO-mutant BLM and a hyper-accumulation of sumoylated BLM. Because multiple homologous recombination proteins are sumoylated in response to replication stress (Xiao et al., 2015), RNF4 depletion might be expected to affect the turnover of multiple factors at stalled forks. Consequently, the phenotypic differences between SM-BLM and RNF4 depletion might also be traced to SUMO-specific modification events of other repair factors.

Cancer cells are often sensitive to the induction of replication stress. The manifestations described here of replication stress under physiologically normal conditions in cells that express a SUMO-mutant BLM suggest that inhibition of BLM sumoylation could make a useful target for chemotherapeutic development. The technical challenge of achieving specific anti-sumoylation activity with a small molecule might be approached through inhibiting BLM’s interaction with the SUMO E3 ligase NSMCE2. More broadly, the genomic and chromatin-associated characteristics of difficult-to-replicate DNA and of the role of sumoylation and other responders to replication stress in facilitating replication fork translocation across these regions are of fundamental importance to our understanding of the process of faithful chromosome duplication.

## Data Availability

The original contributions presented in this study are included in the article and supplementary tables. Further inquiries can be directed to the corresponding author

## References

[B1] Adams M. D. McVey M. Sekelsky J. J. (2003). Drosophila BLM in Double-Strand Break Repair by Synthesis-dependent Strand Annealing. Science 299, 265–267. 10.1126/science.1077198 PubMed Abstract | 10.1126/science.1077198 | Google Scholar 12522255

[B2] Bartos J. D. Wang W. Pike J. E. Bambara R. A. (2006). Mechanisms by Which Bloom Protein Can Disrupt Recombination Intermediates of Okazaki Fragment Maturation. J. Biol. Chem. 281, 32227–32239. 10.1074/jbc.m606310200 PubMed Abstract | 10.1074/jbc.m606310200 | Google Scholar 16950766

[B3] Beresten S. F. Stan R. van Brabant A. J. Ye T. Naureckiene S. Ellis N. A. (1999). Purification of Overexpressed Hexahistidine-Tagged BLM N431 as Oligomeric Complexes. Protein Expr. Purif. 17, 239–248. 10.1006/prep.1999.1135 PubMed Abstract | 10.1006/prep.1999.1135 | Google Scholar 10545272

[B4] Bergink S. Ammon T. Kern M. Schermelleh L. Leonhardt H. Jentsch S. (2013). Role of Cdc48/p97 as a SUMO-Targeted Segregase Curbing Rad51-Rad52 Interaction. Nat. Cell Biol. 15, 526–532. 10.1038/ncb2729 PubMed Abstract | 10.1038/ncb2729 | Google Scholar 23624404

[B5] Bhowmick R. Minocherhomji S. Hickson I. D. (2016). RAD52 Facilitates Mitotic DNA Synthesis Following Replication Stress. Mol. Cell 64, 1117–1126. 10.1016/j.molcel.2016.10.037 PubMed Abstract | 10.1016/j.molcel.2016.10.037 | Google Scholar 27984745

[B6] Bialic M. Coulon V. Drac M. Gostan T. Schwob E. (2015). Analyzing the Dynamics of DNA Replication in Mammalian Cells Using DNA Combing. Methods Mol. Biol. 1300, 67–78. 10.1007/978-1-4939-2596-4_4 PubMed Abstract | 10.1007/978-1-4939-2596-4_4 | Google Scholar 25916705

[B7] Bonner J. N. Choi K. Xue X. Torres N. P. Szakal B. Wei L. (2016). Smc5/6 Mediated Sumoylation of the Sgs1-Top3-Rmi1 Complex Promotes Removal of Recombination Intermediates. Cell Rep. 16, 368–378. 10.1016/j.celrep.2016.06.015 PubMed Abstract | 10.1016/j.celrep.2016.06.015 | Google Scholar 27373152 PMC5051638

[B8] Bythell-Douglas R. Deans A. J. (2021). A Structural Guide to the Bloom Syndrome Complex. Structure 29, 99–113. 10.1016/j.str.2020.11.020 PubMed Abstract | 10.1016/j.str.2020.11.020 | Google Scholar 33357470

[B9] Chaganti R. S. K. Schonberg S. German J. (1974). A Manyfold Increase in Sister Chromatid Exchanges in Bloom's Syndrome Lymphocytes. Proc. Natl. Acad. Sci. U. S. A. 71, 4508–4512. 10.1073/pnas.71.11.4508 PubMed Abstract | 10.1073/pnas.71.11.4508 | Google Scholar 4140506 PMC433916

[B10] Chan K. L. Palmai-Pallag T. Ying S. Hickson I. D. (2009). Replication Stress Induces Sister-Chromatid Bridging at Fragile Site Loci in Mitosis. Nat. Cell Biol. 11, 753–760. 10.1038/ncb1882 PubMed Abstract | 10.1038/ncb1882 | Google Scholar 19465922

[B11] Chan Y. W. West S. C. (2018). A New Class of Ultrafine Anaphase Bridges Generated by Homologous Recombination. Cell Cycle 17, 2101–2109. 10.1080/15384101.2018.1515555 PubMed Abstract | 10.1080/15384101.2018.1515555 | Google Scholar 30253678 PMC6226235

[B12] Cunniff C. Bassetti J. A. Ellis N. A. (2017). Bloom's Syndrome: Clinical Spectrum, Molecular Pathogenesis, and Cancer Predisposition. Mol. Syndromol. 8, 4–23. 10.1159/000452082 PubMed Abstract | 10.1159/000452082 | Google Scholar 28232778 PMC5260600

[B13] Davies S. L. North P. S. Hickson I. D. (2007). Role for BLM in Replication-Fork Restart and Suppression of Origin Firing after Replicative Stress. Nat. Struct. Mol. Biol. 14, 677–679. 10.1038/nsmb1267 PubMed Abstract | 10.1038/nsmb1267 | Google Scholar 17603497

[B14] Drosopoulos W. C. Kosiyatrakul S. T. Schildkraut C. L. (2015). BLM Helicase Facilitates Telomere Replication during Leading Strand Synthesis of Telomeres. J. Cell Biol. 210, 191–208. 10.1083/jcb.201410061 PubMed Abstract | 10.1083/jcb.201410061 | Google Scholar 26195664 PMC4508891

[B15] Dungrawala H. Rose K. L. Bhat K. P. Mohni K. N. Glick G. G. Couch F. B. (2015). The Replication Checkpoint Prevents Two Types of Fork Collapse without Regulating Replisome Stability. Mol. Cell 59, 998–1010. 10.1016/j.molcel.2015.07.030 PubMed Abstract | 10.1016/j.molcel.2015.07.030 | Google Scholar 26365379 PMC4575883

[B16] Eladad S. Ye T.-Z. Hu P. Leversha M. Beresten S. Matunis M. J. (2005). Intra-nuclear Trafficking of the BLM Helicase to DNA Damage-Induced Foci Is Regulated by SUMO Modification. Hum. Mol. Genet. 14, 1351–1365. 10.1093/hmg/ddi145 PubMed Abstract | 10.1093/hmg/ddi145 | Google Scholar 15829507

[B17] Ellis N. A. Groden J. Ye T.-Z. Straughen J. Lennon D. J. Ciocci S. (1995). The Bloom's Syndrome Gene Product Is Homologous to RecQ Helicases. Cell 83, 655–666. 10.1016/0092-8674(95)90105-1 PubMed Abstract | 10.1016/0092-8674(95)90105-1 | Google Scholar 7585968

[B18] Ellis N. Zhu J. Yagle M. K. Yang W.-C. Huang J. Kwako A. (2021). RNF4 Regulates the BLM Helicase in Recovery from Replication Fork Collapse. Front. Genet. 12, 753535. 10.3389/fgene.2021.753535 PubMed Abstract | 10.3389/fgene.2021.753535 | Google Scholar 34868226 PMC8633118

[B19] Flanagan M. Cunniff C. M. (2006). “Bloom Syndrome,” in GeneReviews® [Internet]. Editors Aam M. P. Ardinger H. H. Pagon R. A. Wallace S. E. Bean L. J. H. Gripp K. W. (Seattle, WA: University of Washington), 1993–2022. Google Scholar 20301572

[B20] German J. Archibald R. Bloom D. (1965). Chromosomal Breakage in a Rare and Probably Genetically Determined Syndrome of Man. Science 148, 506–507. 10.1126/science.148.3669.506 PubMed Abstract | 10.1126/science.148.3669.506 | Google Scholar 14263770

[B21] German J. (1997). Bloom's Syndrome. XX. The First 100 Cancers. Cancer Genet. Cytogenet. 93, 100–106. 10.1016/s0165-4608(96)00336-6 PubMed Abstract | 10.1016/s0165-4608(96)00336-6 | Google Scholar 9062585

[B22] Gravel S. Chapman J. R. Magill C. Jackson S. P. (2008). DNA Helicases Sgs1 and BLM Promote DNA Double-Strand Break Resection. Genes Dev. 22, 2767–2772. 10.1101/gad.503108 PubMed Abstract | 10.1101/gad.503108 | Google Scholar 18923075 PMC2569880

[B23] Groden J. Nakamura Y. German J. (1990). Molecular Evidence that Homologous Recombination Occurs in Proliferating Human Somatic Cells. Proc. Natl. Acad. Sci. U.S.A. 87, 4315–4319. 10.1073/pnas.87.11.4315 PubMed Abstract | 10.1073/pnas.87.11.4315 | Google Scholar 1971948 PMC54100

[B24] Hanada K. Budzowska M. Davies S. L. van Drunen E. Onizawa H. Beverloo H. B. (2007). The Structure-specific Endonuclease Mus81 Contributes to Replication Restart by Generating Double-Strand DNA Breaks. Nat. Struct. Mol. Biol. 14, 1096–1104. 10.1038/nsmb1313 PubMed Abstract | 10.1038/nsmb1313 | Google Scholar 17934473

[B25] Hay R. T. (2005). Sumo. Mol. Cell 18, 1–12. 10.1016/j.molcel.2005.03.012 PubMed Abstract | 10.1016/j.molcel.2005.03.012 | Google Scholar 15808504

[B26] Killen M. W. Stults D. M. Adachi N. Hanakahi L. Pierce A. J. (2009). Loss of Bloom Syndrome Protein Destabilizes Human Gene Cluster Architecture. Hum. Mol. Genet. 18, 3417–3428. 10.1093/hmg/ddp282 PubMed Abstract | 10.1093/hmg/ddp282 | Google Scholar 19542097

[B27] Li W. Kim S.-M. Lee J. Dunphy W. G. (2004). Absence of BLM Leads to Accumulation of Chromosomal DNA Breaks during Both Unperturbed and Disrupted S Phases. J. Cell Biol. 165, 801–812. 10.1083/jcb.200402095 PubMed Abstract | 10.1083/jcb.200402095 | Google Scholar 15197177 PMC2172405

[B28] Lönn U. Lönn S. Nylen U. Winblad G. German J. (1990). An Abnormal Profile of DNA Replication Intermediates in Bloom's Syndrome. Cancer Res. 50, 3141–3145. PubMed Abstract | Google Scholar 2110504

[B29] Luo G. Santoro I. M. McDaniel L. D. Nishijima I. Mills M. Youssoufian H. (2000). Cancer Predisposition Caused by Elevated Mitotic Recombination in Bloom Mice. Nat. Genet. 26, 424–429. 10.1038/82548 PubMed Abstract | 10.1038/82548 | Google Scholar 11101838

[B30] Meetei A. R. Sechi S. Wallisch M. Yang D. Young M. K. Joenje H. (2003). A Multiprotein Nuclear Complex Connects Fanconi Anemia and Bloom Syndrome. Mol. Cell Biol. 23, 3417–3426. 10.1128/mcb.23.10.3417-3426.2003 PubMed Abstract | 10.1128/mcb.23.10.3417-3426.2003 | Google Scholar 12724401 PMC164758

[B31] Menolfi D. Delamarre A. Lengronne A. Pasero P. Branzei D. (2015). Essential Roles of the Smc5/6 Complex in Replication through Natural Pausing Sites and Endogenous DNA Damage Tolerance. Mol. Cell 60, 835–846. 10.1016/j.molcel.2015.10.023 PubMed Abstract | 10.1016/j.molcel.2015.10.023 | Google Scholar 26698660 PMC4691243

[B32] Mijic S. Zellweger R. Chappidi N. Berti M. Jacobs K. Mutreja K. (2017). Replication Fork Reversal Triggers Fork Degradation in BRCA2-Defective Cells. Nat. Commun. 8, 859. 10.1038/s41467-017-01164-5 PubMed Abstract | 10.1038/s41467-017-01164-5 | Google Scholar 29038466 PMC5643541

[B33] Minocherhomji S. Ying S. Bjerregaard V. A. Bursomanno S. Aleliunaite A. Wu W. (2015). Replication Stress Activates DNA Repair Synthesis in Mitosis. Nature 528, 286–290. 10.1038/nature16139 PubMed Abstract | 10.1038/nature16139 | Google Scholar 26633632

[B34] Nimonkar A. V. Genschel J. Kinoshita E. Polaczek P. Campbell J. L. Wyman C. (2011). BLM-DNA2-RPA-MRN and EXO1-BLM-RPA-MRN Constitute Two DNA End Resection Machineries for Human DNA Break Repair. Genes Dev. 25, 350–362. 10.1101/gad.2003811 PubMed Abstract | 10.1101/gad.2003811 | Google Scholar 21325134 PMC3042158

[B35] Ouyang K. J. Woo L. L. Zhu J. Huo D. Matunis M. J. Ellis N. A. (2009). SUMO Modification Regulates BLM and RAD51 Interaction at Damaged Replication Forks. PLoS Biol. 7, e1000252. 10.1371/journal.pbio.1000252 PubMed Abstract | 10.1371/journal.pbio.1000252 | Google Scholar 19956565 PMC2779653

[B36] Ouyang K. J. Yagle M. K. Matunis M. J. Ellis N. A. (2013). BLM SUMOylation Regulates ssDNA Accumulation at Stalled Replication Forks. Front. Genet. 4, 167. 10.3389/fgene.2013.00167 PubMed Abstract | 10.3389/fgene.2013.00167 | Google Scholar 24027577 PMC3761158

[B37] Peng X. P. Lim S. Li S. Marjavaara L. Chabes A. Zhao X. (2018). Acute Smc5/6 Depletion Reveals its Primary Role in rDNA Replication by Restraining Recombination at Fork Pausing Sites. PLoS Genet. 14, e1007129. 10.1371/journal.pgen.1007129 PubMed Abstract | 10.1371/journal.pgen.1007129 | Google Scholar 29360860 PMC5779651

[B38] Petermann E. Orta M. L. Issaeva N. Schultz N. Helleday T. (2010). Hydroxyurea-stalled Replication Forks Become Progressively Inactivated and Require Two Different RAD51-Mediated Pathways for Restart and Repair. Mol. Cell 37, 492–502. 10.1016/j.molcel.2010.01.021 PubMed Abstract | 10.1016/j.molcel.2010.01.021 | Google Scholar 20188668 PMC2958316

[B39] Pond K. W. de Renty C. Yagle M. K. Ellis N. A. (2019). Rescue of Collapsed Replication Forks Is Dependent on NSMCE2 to Prevent Mitotic DNA Damage. PLoS Genet. 15, e1007942. 10.1371/journal.pgen.1007942 PubMed Abstract | 10.1371/journal.pgen.1007942 | Google Scholar 30735491 PMC6383951

[B40] Rao V. A. Conti C. Guirouilh-Barbat J. Nakamura A. Miao Z.-H. Davies S. L. (2007). Endogenous γ-H2AX-ATM-Chk2 Checkpoint Activation in Bloom's Syndrome Helicase-Deficient Cells Is Related to DNA Replication Arrested Forks. Mol. Cancer Res. 5, 713–724. 10.1158/1541-7786.mcr-07-0028 PubMed Abstract | 10.1158/1541-7786.mcr-07-0028 | Google Scholar 17634426

[B41] Raynard S. Bussen W. Sung P. (2006). A Double Holliday Junction Dissolvasome Comprising BLM, Topoisomerase IIIα, and BLAP75. J. Biol. Chem. 281, 13861–13864. 10.1074/jbc.c600051200 PubMed Abstract | 10.1074/jbc.c600051200 | Google Scholar 16595695

[B42] Ruchert J. M. Brady M. M. McMahan S. Lacey K. J. Latta L. C. Sekelsky J. (2022). Blm Helicase Facilitates Rapid Replication of Repetitive DNA Sequences in Early Drosophila Development. Genetics 220, iyab169. 10.1093/genetics/iyab169 PubMed Abstract | 10.1093/genetics/iyab169 | Google Scholar 34849849 PMC8733427

[B43] Schlacher K. Christ N. Siaud N. Egashira A. Wu H. Jasin M. (2011). Double-strand Break Repair-independent Role for BRCA2 in Blocking Stalled Replication Fork Degradation by MRE11. Cell 145, 529–542. 10.1016/j.cell.2011.03.041 PubMed Abstract | 10.1016/j.cell.2011.03.041 | Google Scholar 21565612 PMC3261725

[B44] Sfeir A. Kosiyatrakul S. T. Hockemeyer D. MacRae S. L. Karlseder J. Schildkraut C. L. (2009). Mammalian Telomeres Resemble Fragile Sites and Require TRF1 for Efficient Replication. Cell 138, 90–103. 10.1016/j.cell.2009.06.021 PubMed Abstract | 10.1016/j.cell.2009.06.021 | Google Scholar 19596237 PMC2723738

[B45] Sharma S. Sommers J. A. Wu L. Bohr V. A. Hickson I. D. Brosh R. M. Jr. (2004). Stimulation of Flap Endonuclease-1 by the Bloom's Syndrome Protein. J. Biol. Chem. 279, 9847–9856. 10.1074/jbc.m309898200 PubMed Abstract | 10.1074/jbc.m309898200 | Google Scholar 14688284

[B46] Shastri V. M. Subramanian V. Schmidt K. H. (2021). A Novel Cell-Cycle-Regulated Interaction of the Bloom Syndrome Helicase BLM with Mcm6 Controls Replication-Linked Processes. Nucleic Acids Res. 49, 8699–8713. 10.1093/nar/gkab663 PubMed Abstract | 10.1093/nar/gkab663 | Google Scholar 34370039 PMC8421143

[B47] Shima H. Suzuki H. Sun J. Kono K. Shi L. Kinomura A. (2013). Activation of the SUMO Modification System Is Required for the Accumulation of RAD51 at Sites of DNA Damage. J. Cell Sci. 126, 5284–5292. 10.1242/jcs.133744 PubMed Abstract | 10.1242/jcs.133744 | Google Scholar 24046452

[B48] Shorrocks A.-M. K. Jones S. E. Tsukada K. Morrow C. A. Belblidia Z. Shen J. (2021). The Bloom Syndrome Complex Senses RPA-Coated Single-Stranded DNA to Restart Stalled Replication Forks. Nat. Commun. 12, 585. 10.1038/s41467-020-20818-5 PubMed Abstract | 10.1038/s41467-020-20818-5 | Google Scholar 33500419 PMC7838300

[B49] Sidorova J. M. Li N. Schwartz D. C. Folch A. Monnat Jr R. J. Jr. (2009). Microfluidic-assisted Analysis of Replicating DNA Molecules. Nat. Protoc. 4, 849–861. 10.1038/nprot.2009.54 PubMed Abstract | 10.1038/nprot.2009.54 | Google Scholar 19444242 PMC2762429

[B50] Soniat M. M. Myler L. R. Kuo H.-C. Paull T. T. Finkelstein I. J. (2019). RPA Phosphorylation Inhibits DNA Resection. Mol. Cell 75, 145–153. 10.1016/j.molcel.2019.05.005 PubMed Abstract | 10.1016/j.molcel.2019.05.005 | Google Scholar 31153714 PMC6625828

[B51] Spies J. Lukas C. Somyajit K. Rask M.-B. Lukas J. Neelsen K. J. (2019). 53BP1 Nuclear Bodies Enforce Replication Timing at Under-replicated DNA to Limit Heritable DNA Damage. Nat. Cell Biol. 21, 487–497. 10.1038/s41556-019-0293-6 PubMed Abstract | 10.1038/s41556-019-0293-6 | Google Scholar 30804506

[B52] Técher H. Koundrioukoff S. Azar D. Wilhelm T. Carignon S. Brison O. (2013). Replication Dynamics: Biases and Robustness of DNA Fiber Analysis. J. Mol. Biol. 425, 4845–4855. 10.1016/j.jmb.2013.03.040 PubMed Abstract | 10.1016/j.jmb.2013.03.040 | Google Scholar 23557832

[B53] Vesela E. Chroma K. Turi Z. Mistrik M. (2017). Common Chemical Inductors of Replication Stress: Focus on Cell‐Based Studies. Biomolecules 7, 19. 10.3390/biom7010019 PubMed Abstract | 10.3390/biom7010019 | Google Scholar

[B54] Whelan D. R. Rothenberg E. (2021). Super-resolution Mapping of Cellular Double-Strand Break Resection Complexes during Homologous Recombination. Proc. Natl. Acad. Sci. U.S.A. 118, e2021963118. 10.1073/pnas.2021963118 PubMed Abstract | 10.1073/pnas.2021963118 | Google Scholar 33707212 PMC7980414

[B55] Wu L. Bachrati C. Z. Ou J. Xu C. Yin J. Chang M. (2006). BLAP75/RMI1 Promotes the BLM-dependent Dissolution of Homologous Recombination Intermediates. Proc. Natl. Acad. Sci. U.S.A. 103, 4068–4073. 10.1073/pnas.0508295103 PubMed Abstract | 10.1073/pnas.0508295103 | Google Scholar 16537486 PMC1449647

[B56] Wu L. Hickson I. D. (2003). The Bloom's Syndrome Helicase Suppresses Crossing over during Homologous Recombination. Nature 426, 870–874. 10.1038/nature02253 PubMed Abstract | 10.1038/nature02253 | Google Scholar 14685245

[B57] Xiao Z. Chang J.-G. Hendriks I. A. Sigurðsson J. O. Olsen J. V. Vertegaal A. C. O. (2015). System-wide Analysis of SUMOylation Dynamics in Response to Replication Stress Reveals Novel Small Ubiquitin-like Modified Target Proteins and Acceptor Lysines Relevant for Genome Stability. Mol. Cell. Proteomics 14, 1419–1434. 10.1074/mcp.o114.044792 PubMed Abstract | 10.1074/mcp.o114.044792 | Google Scholar 25755297 PMC4424410

[B58] Yankiwski V. Marciniak R. A. Guarente L. Neff N. F. (2000). Nuclear Structure in Normal and Bloom Syndrome Cells. Proc. Natl. Acad. Sci. U.S.A. 97, 5214–5219. 10.1073/pnas.090525897 PubMed Abstract | 10.1073/pnas.090525897 | Google Scholar 10779560 PMC25808

[B59] Zellweger R. Dalcher D. Mutreja K. Berti M. Schmid J. A. Herrador R. (2015). Rad51-mediated Replication Fork Reversal Is a Global Response to Genotoxic Treatments in Human Cells. J. Cell Biol. 208, 563–579. 10.1083/jcb.201406099 PubMed Abstract | 10.1083/jcb.201406099 | Google Scholar 25733714 PMC4347635

[B60] Zeman M. K. Cimprich K. A. (2014). Causes and Consequences of Replication Stress. Nat. Cell Biol. 16, 2–9. 10.1038/ncb2897 PubMed Abstract | 10.1038/ncb2897 | Google Scholar 24366029 PMC4354890

[B61] Zhang R. Sengupta S. Yang Q. Linke S. P. Yanaihara N. Bradsher J. (2005). BLM Helicase Facilitates Mus81 Endonuclease Activity in Human Cells. Cancer Res. 65, 2526–2531. 10.1158/0008-5472.can-04-2421 PubMed Abstract | 10.1158/0008-5472.can-04-2421 | Google Scholar 15805243

[B62] Zhu J. Zhu S. Guzzo C. M. Ellis N. A. Sung K. S. Choi C. Y. (2008). Small Ubiquitin-Related Modifier (SUMO) Binding Determines Substrate Recognition and Paralog-Selective SUMO Modification. J. Biol. Chem. 283, 29405–29415. 10.1074/jbc.m803632200 PubMed Abstract | 10.1074/jbc.m803632200 | Google Scholar 18708356 PMC2570875

[B63] Zimmermann M. Kibe T. Kabir S. de Lange T. (2014). TRF1 Negotiates TTAGGG Repeat-Associated Replication Problems by Recruiting the BLM Helicase and the TPP1/POT1 Repressor of ATR Signaling. Genes Dev. 28, 2477–2491. 10.1101/gad.251611.114 PubMed Abstract | 10.1101/gad.251611.114 | Google Scholar 25344324 PMC4233241

