# Supplementary material for: BLM Sumoylation Is Required for Replication Stability and Normal Fork Velocity During DNA Replication
Source: Front Mol Biosci. 2022 Jul 1;9:875102. doi: 10.3389/fmolb.2022.875102 (PMC9284272; doi:10.3389/fmolb.2022.875102)
Supplement: Supplementary file 1 [file Table1.DOCX]

**Supplementary table**: Statistics for Figure 1; Two-Way ANOVA multiple comparison from GraphPad PRISM.

| Within each column, compare rows (simple effects within columns) | | | | | | | | |
| --- | --- | --- | --- | --- | --- | --- | --- | --- |
|  |  |  |  |  |  |  |  |  |
| Number of families | 3 |  |  |  |  |  |  |  |
| Number of comparisons per family | 15 |  |  |  |  |  |  |  |
| Alpha | 0.05 |  |  |  |  |  |  |  |
|  |  |  |  |  |  |  |  |  |
| Tukey's multiple comparisons test | Mean Diff. | 95.00% CI of diff. | Significant? | Summary | Adjusted P Value |  |  |  |
|  |  |  |  |  |  |  |  |  |
| **Fork restart** |  |  |  |  |  |  |  |  |
| BLM+ NT vs. BLM+ HU | 1.365 | -6.952 to 9.682 | No | ns | 0.9946 |  |  |  |
| BLM+ NT vs. SM-BLM NT | 1.577 | -6.740 to 9.894 | No | ns | 0.9895 |  |  |  |
| BLM+ NT vs. SM-BLM HU | 15.53 | 7.215 to 23.85 | Yes | *** | 0.0002 |  |  |  |
| BLM+ NT vs. BLM- NT | 5.920 | -2.397 to 14.24 | No | ns | 0.2591 |  |  |  |
| BLM+ NT vs. BLM- HU | 19.51 | 11.20 to 27.83 | Yes | **** | <0.0001 |  |  |  |
| BLM+ HU vs. SM-BLM NT | 0.2123 | -8.105 to 8.529 | No | ns | >0.9999 |  |  |  |
| BLM+ HU vs. SM-BLM HU | 14.17 | 5.850 to 22.48 | Yes | *** | 0.0005 |  |  |  |
| BLM+ HU vs. BLM- NT | 4.555 | -3.762 to 12.87 | No | ns | 0.5247 |  |  |  |
| BLM+ HU vs. BLM- HU | 18.15 | 9.832 to 26.47 | Yes | **** | <0.0001 |  |  |  |
| SM-BLM NT vs. SM-BLM HU | 13.95 | 5.638 to 22.27 | Yes | *** | 0.0006 |  |  |  |
| SM-BLM NT vs. BLM- NT | 4.343 | -3.974 to 12.66 | No | ns | 0.5731 |  |  |  |
| SM-BLM NT vs. BLM- HU | 17.94 | 9.620 to 26.25 | Yes | **** | <0.0001 |  |  |  |
| SM-BLM HU vs. BLM- NT | -9.612 | -17.93 to -1.295 | Yes | * | 0.0183 |  |  |  |
| SM-BLM HU vs. BLM- HU | 3.982 | -4.335 to 12.30 | No | ns | 0.6558 |  |  |  |
| BLM- NT vs. BLM- HU | 13.59 | 5.277 to 21.91 | Yes | *** | 0.0007 |  |  |  |
|  |  |  |  |  |  |  |  |  |
| **collapsed forks** |  |  |  |  |  |  |  |  |
| BLM+ NT vs. BLM+ HU | -1.558 | -9.875 to 6.759 | No | ns | 0.9901 |  |  |  |
| BLM+ NT vs. SM-BLM NT | -1.771 | -10.09 to 6.546 | No | ns | 0.9824 |  |  |  |
| BLM+ NT vs. SM-BLM HU | -14.61 | -22.93 to -6.291 | Yes | *** | 0.0003 |  |  |  |
| BLM+ NT vs. BLM- NT | -4.661 | -12.98 to 3.656 | No | ns | 0.5009 |  |  |  |
| BLM+ NT vs. BLM- HU | -13.60 | -21.92 to -5.285 | Yes | *** | 0.0007 |  |  |  |
| BLM+ HU vs. SM-BLM NT | -0.2131 | -8.530 to 8.104 | No | ns | >0.9999 |  |  |  |
| BLM+ HU vs. SM-BLM HU | -13.05 | -21.37 to -4.734 | Yes | ** | 0.0011 |  |  |  |
| BLM+ HU vs. BLM- NT | -3.103 | -11.42 to 5.214 | No | ns | 0.8379 |  |  |  |
| BLM+ HU vs. BLM- HU | -12.04 | -20.36 to -3.727 | Yes | ** | 0.0026 |  |  |  |
| SM-BLM NT vs. SM-BLM HU | -12.84 | -21.15 to -4.521 | Yes | ** | 0.0014 |  |  |  |
| SM-BLM NT vs. BLM- NT | -2.890 | -11.21 to 5.427 | No | ns | 0.8733 |  |  |  |
| SM-BLM NT vs. BLM- HU | -11.83 | -20.15 to -3.514 | Yes | ** | 0.0031 |  |  |  |
| SM-BLM HU vs. BLM- NT | 9.948 | 1.631 to 18.26 | Yes | * | 0.0140 |  |  |  |
| SM-BLM HU vs. BLM- HU | 1.007 | -7.310 to 9.324 | No | ns | 0.9987 |  |  |  |
| BLM- NT vs. BLM- HU | -8.941 | -17.26 to -0.6237 | Yes | * | 0.0310 |  |  |  |
|  |  |  |  |  |  |  |  |  |
| **new origins** |  |  |  |  |  |  |  |  |
| BLM+ NT vs. BLM+ HU | 0.1930 | -8.124 to 8.510 | No | ns | >0.9999 |  |  |  |
| BLM+ NT vs. SM-BLM NT | 0.1939 | -8.123 to 8.511 | No | ns | >0.9999 |  |  |  |
| BLM+ NT vs. SM-BLM HU | -0.9235 | -9.241 to 7.394 | No | ns | 0.9991 |  |  |  |
| BLM+ NT vs. BLM- NT | -1.259 | -9.576 to 7.058 | No | ns | 0.9963 |  |  |  |
| BLM+ NT vs. BLM- HU | -5.913 | -14.23 to 2.405 | No | ns | 0.2602 |  |  |  |
| BLM+ HU vs. SM-BLM NT | 0.0008235 | -8.316 to 8.318 | No | ns | >0.9999 |  |  |  |
| BLM+ HU vs. SM-BLM HU | -1.117 | -9.434 to 7.201 | No | ns | 0.9979 |  |  |  |
| BLM+ HU vs. BLM- NT | -1.452 | -9.769 to 6.865 | No | ns | 0.9928 |  |  |  |
| BLM+ HU vs. BLM- HU | -6.106 | -14.42 to 2.211 | No | ns | 0.2317 |  |  |  |
| SM-BLM NT vs. SM-BLM HU | -1.117 | -9.434 to 7.200 | No | ns | 0.9979 |  |  |  |
| SM-BLM NT vs. BLM- NT | -1.453 | -9.770 to 6.864 | No | ns | 0.9928 |  |  |  |
| SM-BLM NT vs. BLM- HU | -6.106 | -14.42 to 2.211 | No | ns | 0.2316 |  |  |  |
| SM-BLM HU vs. BLM- NT | -0.3354 | -8.652 to 7.982 | No | ns | >0.9999 |  |  |  |
| SM-BLM HU vs. BLM- HU | -4.989 | -13.31 to 3.328 | No | ns | 0.4297 |  |  |  |
| BLM- NT vs. BLM- HU | -4.654 | -12.97 to 3.663 | No | ns | 0.5025 |  |  |  |
|  |  |  |  |  |  |  |  |  |
|  |  |  |  |  |  |  |  |  |
| **Test details** | **Mean 1** | **Mean 2** | **Mean Diff.** | **SE of diff.** | **N1** | **N2** | **q** | **DF** |
|  |  |  |  |  |  |  |  |  |
| **Fork restart** |  |  |  |  |  |  |  |  |
| BLM+ NT vs. BLM+ HU | 95.71 | 94.34 | 1.365 | 2.617 | 2 | 2 | 0.7375 | 18.00 |
| BLM+ NT vs. SM-BLM NT | 95.71 | 94.13 | 1.577 | 2.617 | 2 | 2 | 0.8522 | 18.00 |
| BLM+ NT vs. SM-BLM HU | 95.71 | 80.17 | 15.53 | 2.617 | 2 | 2 | 8.393 | 18.00 |
| BLM+ NT vs. BLM- NT | 95.71 | 89.79 | 5.920 | 2.617 | 2 | 2 | 3.199 | 18.00 |
| BLM+ NT vs. BLM- HU | 95.71 | 76.19 | 19.51 | 2.617 | 2 | 2 | 10.55 | 18.00 |
| BLM+ HU vs. SM-BLM NT | 94.34 | 94.13 | 0.2123 | 2.617 | 2 | 2 | 0.1147 | 18.00 |
| BLM+ HU vs. SM-BLM HU | 94.34 | 80.17 | 14.17 | 2.617 | 2 | 2 | 7.656 | 18.00 |
| BLM+ HU vs. BLM- NT | 94.34 | 89.79 | 4.555 | 2.617 | 2 | 2 | 2.461 | 18.00 |
| BLM+ HU vs. BLM- HU | 94.34 | 76.19 | 18.15 | 2.617 | 2 | 2 | 9.808 | 18.00 |
| SM-BLM NT vs. SM-BLM HU | 94.13 | 80.17 | 13.95 | 2.617 | 2 | 2 | 7.541 | 18.00 |
| SM-BLM NT vs. BLM- NT | 94.13 | 89.79 | 4.343 | 2.617 | 2 | 2 | 2.347 | 18.00 |
| SM-BLM NT vs. BLM- HU | 94.13 | 76.19 | 17.94 | 2.617 | 2 | 2 | 9.693 | 18.00 |
| SM-BLM HU vs. BLM- NT | 80.17 | 89.79 | -9.612 | 2.617 | 2 | 2 | 5.194 | 18.00 |
| SM-BLM HU vs. BLM- HU | 80.17 | 76.19 | 3.982 | 2.617 | 2 | 2 | 2.152 | 18.00 |
| BLM- NT vs. BLM- HU | 89.79 | 76.19 | 13.59 | 2.617 | 2 | 2 | 7.346 | 18.00 |
|  |  |  |  |  |  |  |  |  |
| **collapsed forks** |  |  |  |  |  |  |  |  |
| BLM+ NT vs. BLM+ HU | 3.559 | 5.117 | -1.558 | 2.617 | 2 | 2 | 0.8418 | 18.00 |
| BLM+ NT vs. SM-BLM NT | 3.559 | 5.330 | -1.771 | 2.617 | 2 | 2 | 0.9570 | 18.00 |
| BLM+ NT vs. SM-BLM HU | 3.559 | 18.17 | -14.61 | 2.617 | 2 | 2 | 7.894 | 18.00 |
| BLM+ NT vs. BLM- NT | 3.559 | 8.220 | -4.661 | 2.617 | 2 | 2 | 2.519 | 18.00 |
| BLM+ NT vs. BLM- HU | 3.559 | 17.16 | -13.60 | 2.617 | 2 | 2 | 7.350 | 18.00 |
| BLM+ HU vs. SM-BLM NT | 5.117 | 5.330 | -0.2131 | 2.617 | 2 | 2 | 0.1152 | 18.00 |
| BLM+ HU vs. SM-BLM HU | 5.117 | 18.17 | -13.05 | 2.617 | 2 | 2 | 7.052 | 18.00 |
| BLM+ HU vs. BLM- NT | 5.117 | 8.220 | -3.103 | 2.617 | 2 | 2 | 1.677 | 18.00 |
| BLM+ HU vs. BLM- HU | 5.117 | 17.16 | -12.04 | 2.617 | 2 | 2 | 6.508 | 18.00 |
| SM-BLM NT vs. SM-BLM HU | 5.330 | 18.17 | -12.84 | 2.617 | 2 | 2 | 6.937 | 18.00 |
| SM-BLM NT vs. BLM- NT | 5.330 | 8.220 | -2.890 | 2.617 | 2 | 2 | 1.562 | 18.00 |
| SM-BLM NT vs. BLM- HU | 5.330 | 17.16 | -11.83 | 2.617 | 2 | 2 | 6.393 | 18.00 |
| SM-BLM HU vs. BLM- NT | 18.17 | 8.220 | 9.948 | 2.617 | 2 | 2 | 5.376 | 18.00 |
| SM-BLM HU vs. BLM- HU | 18.17 | 17.16 | 1.007 | 2.617 | 2 | 2 | 0.5441 | 18.00 |
| BLM- NT vs. BLM- HU | 8.220 | 17.16 | -8.941 | 2.617 | 2 | 2 | 4.831 | 18.00 |
|  |  |  |  |  |  |  |  |  |
| **new origins** |  |  |  |  |  |  |  |  |
| BLM+ NT vs. BLM+ HU | 0.7347 | 0.5416 | 0.1930 | 2.617 | 2 | 2 | 0.1043 | 18.00 |
| BLM+ NT vs. SM-BLM NT | 0.7347 | 0.5408 | 0.1939 | 2.617 | 2 | 2 | 0.1048 | 18.00 |
| BLM+ NT vs. SM-BLM HU | 0.7347 | 1.658 | -0.9235 | 2.617 | 2 | 2 | 0.4991 | 18.00 |
| BLM+ NT vs. BLM- NT | 0.7347 | 1.994 | -1.259 | 2.617 | 2 | 2 | 0.6803 | 18.00 |
| BLM+ NT vs. BLM- HU | 0.7347 | 6.647 | -5.913 | 2.617 | 2 | 2 | 3.195 | 18.00 |
| BLM+ HU vs. SM-BLM NT | 0.5416 | 0.5408 | 0.0008235 | 2.617 | 2 | 2 | 0.0004450 | 18.00 |
| BLM+ HU vs. SM-BLM HU | 0.5416 | 1.658 | -1.117 | 2.617 | 2 | 2 | 0.6034 | 18.00 |
| BLM+ HU vs. BLM- NT | 0.5416 | 1.994 | -1.452 | 2.617 | 2 | 2 | 0.7846 | 18.00 |
| BLM+ HU vs. BLM- HU | 0.5416 | 6.647 | -6.106 | 2.617 | 2 | 2 | 3.299 | 18.00 |
| SM-BLM NT vs. SM-BLM HU | 0.5408 | 1.658 | -1.117 | 2.617 | 2 | 2 | 0.6038 | 18.00 |
| SM-BLM NT vs. BLM- NT | 0.5408 | 1.994 | -1.453 | 2.617 | 2 | 2 | 0.7851 | 18.00 |
| SM-BLM NT vs. BLM- HU | 0.5408 | 6.647 | -6.106 | 2.617 | 2 | 2 | 3.300 | 18.00 |
| SM-BLM HU vs. BLM- NT | 1.658 | 1.994 | -0.3354 | 2.617 | 2 | 2 | 0.1813 | 18.00 |
| SM-BLM HU vs. BLM- HU | 1.658 | 6.647 | -4.989 | 2.617 | 2 | 2 | 2.696 | 18.00 |
| BLM- NT vs. BLM- HU | 1.994 | 6.647 | -4.654 | 2.617 | 2 | 2 | 2.515 | 18.00 |
